# An Integrated Approach for the Environmental Characterization of a Wide Potentially Contaminated Area in Southern Italy

**DOI:** 10.3390/ijerph14070693

**Published:** 2017-06-27

**Authors:** Daniela Ducci, Stefano Albanese, Lorenzo Boccia, Egidio Celentano, Elena Cervelli, Alfonso Corniello, Anna Crispo, Benedetto De Vivo, Paolo Iodice, Carmela Langella, Annamaria Lima, Maurizio Manno, Mario Palladino, Stefania Pindozzi, Marina Rigillo, Nunzio Romano, Mariangela Sellerino, Adolfo Senatore, Giuseppe Speranza, Nunzio Fiorentino, Massimo Fagnano

**Affiliations:** 1 Dipartimento di Ingegneria Civile Edile e Ambientale, University of Naples Federico II, 80125 Naples, Italy; daniela@unina.it (D.D.); corniell@unina.it (A.C.); 2 Dipartimento di Scienze della Terra, dell’Ambiente e delle Risorse, University of Naples Federico II, 80138 Naples, Italy; stefano.albanese@unina.it (S.A.); bdevivo@unina.it (B.D.V.); anlima@unina.it (A.L.); 3 Dipartimento di Agraria, University of Naples Federico II, 80055 Portici (NA), Italy; lorenzo.boccia@unina.it (L.B.); mario.palladino@unina.it (M.P.); stefania.pindozzi@unina.it (S.P.); nunzio.romano@unina.it (N.R.); giuseppe.speranza70@gmail.com (G.S.); nunzio.fiorentino@unina.it (N.F.); massimo.fagnano@unina.it (M.F.); 4 Agenzia Regionale Sanitaria Della Campania, 80143 Naples, Italy; e.celentano@istitutotumori.na.it; 5 Centro Interdipartimentale Ricerca Ambiente, University of Naples Federico II, 80138 Naples, Italy; elena.cervelli@unina.it; 6 Epidemiology Unit, National Cancer Institute, “G. Pascale” Foundation, 80131 Naples, Italy; anna.crispo@tin.it; 7 Dipartimento di Ingegneria Industriale, University of Naples Federico II, 80125 Naples, Italy; paolo.iodice@unina.it (P.I.); adolfo.senatore@unina.it (A.S.); 8 Dipartimento di Sanità Pubblica, University of Naples Federico II, 80131 Naples, Italy; ela.langella@hotmail.it (C.L.); maurizio.manno@unina.it (M.M.); 9 Dipartimento di Architettura, University of Naples Federico II, 80134 Naples, Italy; marina.rigillo@unina.it

**Keywords:** air pollution, soil hydraulic parameters, geochemical characterization of soils, aquifer vulnerability to contamination, health assessment, multi-criteria environmental analysis

## Abstract

This paper deals with the environmental characterization of a large and densely populated area, with a poor reputation for contamination, considering the contribution of environmental features (air, soil, soil hydraulic and groundwater) and the potential effects on human health. The use of Geographic Information System (GIS) has made possible a georeferenced inventory and, by overlaying environmental information, an operational synthesis of comprehensive environmental conditions. The cumulative effects on environmental features were evaluated, taking into account superposition effects, by means of the Spatial MultiCriteria Decision Analysis (S-MCDA). The application of the S-MCDA for converging the combination of heterogeneous factors, related to soil, land and water, deeply studied by heterogeneous groups of experts, constitutes the novelty of the paper. The results confirmed an overall higher potential of exposure to contaminants in the environment and higher mortality rates in the study area for some tumours, but hospital admissions for tumours were generally similar to the regional trend. Besides, mortality data may be strictly dependent on the poor socioeconomic conditions, quality of therapy and a lack of welfare in the area relative to the rest of Italy. Finally, as regards the possible relationship between presence of contaminants in the environment and health conditions of the population no definite conclusions can be drawn, although the present study encourages the use of the new proposed methods, that increase the possibilities for studying the combined effect of more environmental factors.

## 1. Introduction

The Litorale Domizio-Agro Aversano area of the Campania region in southern Italy was recognized as a National Interest Priority Site (NIPS) in 1998, owing to the large number of potentially polluted sites, caused by widespread illegal dumping of industrial and municipal waste [1]. Italian law (L. 426/98 and D. Lgs. 152/06) requires that reclamation activities must be carried out in NIPSs. In 2013, the priority level of the area for reclamation of contaminated areas was downgraded from national to regional by the Italian authorities. This downgrade did not correspond to a decrease of contamination status, but rather to a transfer of competence for the reclamation work, now ascribed to the Campania Region.

In the last decade, widespread burning of illegal waste degraded the reputation of the southern part of the area, which thereof has been called the “Land of Fire” by the media (New York Times, 30 January 2014, page A4 of the New York edition). Although the percentage of land contaminated is low, broadcast journalism frequently frightens the public by identifying the entire area as contaminated. Consequently the appeal of local food products has been reduced and this has thrown the agricultural economy of the region into crisis.

Given the fragmentary background information available for this area, a large number of remediation tools has been considered to reduce the potential environmental and health risks. The rehabilitation strategy for the area and the costs of reclaiming such a large number of sites strongly depend on the accuracy of area characterization, which would allow targeted and localized reclamation actions.

The goal of the present study is to verify the state of the air, soil and water pollution, identifying areas where appropriate reclamation strategies are needed. Achieving these objectives would help the area to return to traditional agricultural use, in order to protect the original rural landscape and guarantee traditional agricultural resource income, now residual or abandoned.

The study has been developed in the framework of the “Implementation of eco-compatible protocols for agricultural soil remediation in Litorale Domizio-Agro Aversano NIPS” (ECOREMED) project (LIFE11/ENV/IT/275), which main aim is the evaluation of different phyto- and bio-remediation protocols at pilot sites scale. The ECOREMED protocol is a standardized process for the application of reclamation techniques using phyto- and bio-remediation.

The first step of this study has been the collection of existing data on the agricultural Litorale Domizio-Agro Aversano area and the building of a geo-database in a GIS environment. Available data have been integrated with new measurements to update knowledge of the environmental factors in the area.

Afterwards, it was carried out an integrated environmental-health characterization of the area, assessing the contribution of different driving forces to the contamination of environmental matrices (air, soil, soil hydraulic and groundwater). A Spatial MultiCriteria Decision Analysis (S-MCDA) integrating the Geographic Information System (GIS) and the MultiCriteria Decision Analysis (MCDA), has been used in this study to sort out the suitability map for the application of phyto-remediation reclamation strategies.

One of the main constraints in the land management policies is the integration of different or conflicting land dynamics interpretations. The S-MCDA approach allows the management of the land system as a complex entity, generally studied according to different approaches based on specific expertise. Some studies report S-MCDA application as a transparent process for choice analysis, open to the community, able to manage the comparison of alternatives and for the easiness of exchange data and information [2,3,4].

The value of the study and thus the novelty consists mainly in the synergetic approach among different scientists and in the uniqueness of the study area in terms of extent (>1500 km^2^) and population density (~1,400,000 inhabitants in 77 municipalities). In short, the cumulative effects on several environmental endpoints were evaluated, considering, by means the S-MCDA technique, various potential in-combination effects [5,6,7,8,9].

As part of the environmental characterization, a set of potential scenarios were developed with the aim of identifying areas suitable for application of the bio-remediation practices. The use of GIS has made possible the overlay of information from studies of different environmental features and operational syntheses of comprehensive environmental conditions.

### Study Area

**Figure 1:**
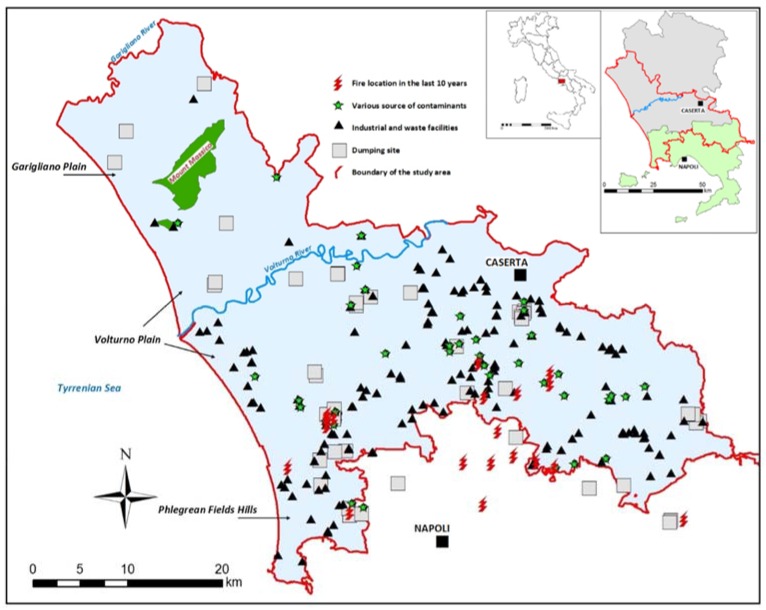
Location of the study area and main sources of contamination. In the square on the top-right corner the provinces of Napoli and Caserta compared with the study area boundaries.

Litorale Domizio-Agro Aversano encompasses the plains of the Garigliano and Volturno rivers and part of the volcanic area of the Phlegrean Fields (Figure 1). Volcanic soils have been beneficial for agricultural development in the study area since Roman times. Local economic development has been historically based on agriculture (including olive groves, vineyards, and fruit trees), livestock raising, and occasionally tourism (along the coastline).

Attention should be focused on the central and southern areas, on the left side of the Volturno river, where diversified human activities coexist, i.e., predominately intensive agriculture and grazing and industrial facilities. In this densely populated area (density almost everywhere is >1000 per km^2^), environmental contamination is attributed to an insufficient sewage network and to legal and illegal dumping of waste, with hazardous consequences for soil and groundwater [10,11,12]. The Garigliano plain, separated from the Volturno plain by Mount Massico, has decidedly less anthropogenic pressure (density is <250 per km^2^). During summer, there is an increase in population because of tourism along the coast of the entire area, which is largely flat except for Mount Massico and the slopes of the Phlegrean Hills.

**Figure 2:**
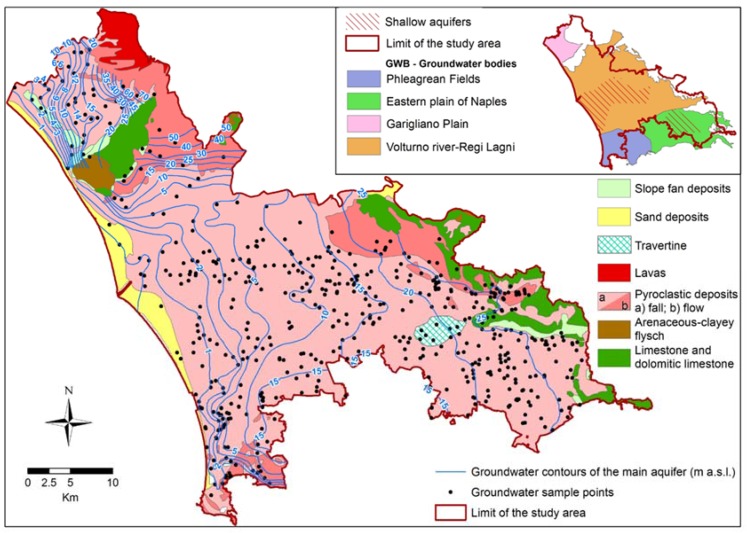
Main hydrogeological features of the study area. In the upper right corner the partition of the study area in groundwater bodies (GWBs).

Indeed, the flat coastal area crossed by the Garigliano and Volturno rivers is a Quaternary tectonic depression filled by alluvial and volcanic deposits. The *graben*, whose subsidence continued over the entire Quaternary, is bounded by the Mesozoic carbonate ridges of the southern Apennines (north and east) and by the Roccamonfina volcano (northeast). There are tuffs and pyroclastic deposits, emitted by the Phlegrean Fields during the Late Pleistocene-Holocene, in the subsoil of the plain and outcrop in the hills of those fields. The area encompasses the whole Garigliano plain groundwater body (GWB) (as defined in the river basin management plans 2015–2021, see http://www.ildistrettoidrograficodellappenninomeridionale.it/), and parts of the GWBs of the plain of the Volturno river—Regi Lagni, the eastern Plain of Naples, and the Phleagrean Fields (Figure 2).

The Garigliano plain GWB (137 km^2^) consists of marine and alluvial deposits, inter-bedded with pyroclastic in its northeast sector, close to the Roccamonfina volcano [13]. The plain of the Volturno river—Regi Lagni GWB (1069 km^2^, 860 km^2^ of which in the study area) includes alluvial, pyroclastic and marine deposits separated by tuffs (Campanian Ignimbrite) in shallow aquifers and the deeper main aquifer [10]. The eastern Plain of Naples GWB (430 km^2^, 220 km^2^ of which in the study area) is constituted approximately by the same deposits, but the tuffs are often absent. The aquifer of the Phleagrean Fields GWB (203 km^2^, 100 km^2^ of which is in the study area) is a succession of pyroclastic beds with different grain sizes and cementation degrees. Groundwater flow is directed everywhere toward the sea and the Garigliano and Volturno rivers (Figure 2).

The climate in the Campania region in general, and over the study area in particular, is affected by noticeable spatial and temporal structures of rainfall variability. Mean annual temperatures range between 10 °C in the mountainous interior to 18 °C in the coastal areas, while rainfall, with a maximum in autumn/winter, varies between 700 mm/y in the eastern part of the region, to 2000 mm/y in the mountains (1920–2010). November is the month with the heaviest rainfalls, and August is typically the driest month. The coldest period is the end of January to the beginning of February.

## 2. Materials and Methods

**Figure 3:**
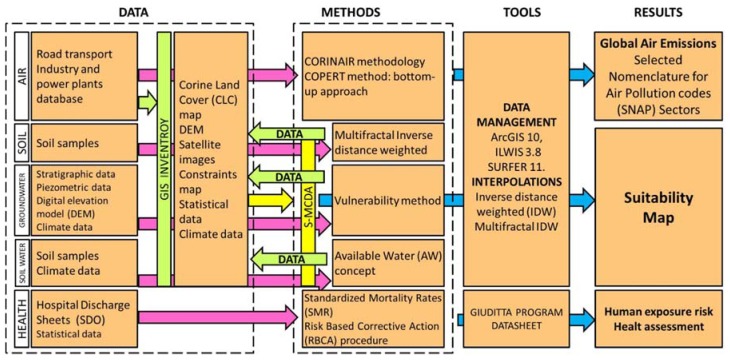
Flow chart of the integrated environmental-health characterization of the study area, via GIS inventory and Spatial MultiCriteria Decision Analysis (S-MCDA). Suitability map refers to areas suitable for bioremediation reclamation techniques.

Figure 3 shows the flow chart of the integrated environmental-health characterization of the study area, with the different phases of the study. The collection of existing data on the agricultural Litorale Domizio-Agro Aversano area was aimed to build a geo-database in a GIS environment. Available data have been integrated with new measurements to update the knowledge of the environmental factors in the area. All data were managed with the software ArcGIS 10, ILWIS 3.8 and SURFER 11.

### 2.1. Total Emissions of Air Pollutants

For environmental characterization, the atmospheric emission inventory of the major air pollutants and the Total Particulate Matter (TPM) in the study area were estimated by a bottom-up method, which focused on the municipal level, examining and involving detailed local data rather than national information, and considering a spatial distribution of emission levels with communal disaggregation. These emission inventories constituted a technological and territorial database, which located all air pollution sources (point and non-point) with varying spatial disaggregation (national, regional and urban), and characterized the quantity and type of pollutant. At the European level, the atmospheric emission inventories are built in compliance with the COoRdination INformation AIR (CORINAIR) methodology, which provides guidance on estimating emissions from both anthropogenic and natural emission sources [14]. The activities responsible for the emissions were classified in sectors according to the Selected Nomenclature for Air Pollution codes (SNAP version 1997).

The emission levels of the main air pollutants and TPMs were appraised for fuel combustion and non-combustion sources in the following anthropogenic activity sectors: transformation (comprising electricity and heat generation, petroleum refineries, and similar transformation industries); other industrial sectors (including iron and steel, chemical, and nonferrous plants); road transport and other activity sectors (including commerce, agriculture, and residential).

Among the above emission sources, two groups were given particular attention, mobile (the road transport sector) and point (mostly industry and power plants) sources. In many European nations, these sources constitute the main share of anthropogenic emissions of the air pollutants considered in this paper.

Pollutant emissions from the most significant industries were assessed using actual measurements. Since the large industrial sources are ruled by regulations and licenses, these factories are demanded to disclose their annual emission levels. Therefore, in order to acquire direct measurements for the pertinent point sources (namely the emission levels for each observed stack), surveys were performed in the main industries existing in the area under investigation, which afterward measure and report the emission levels of the chief air pollutants (CO, NOx, VOC and PM_10_) produced annually. These factories mainly comprised industrial boilers and power plants fuelled by liquefied petroleum gas, diesel and natural gas.

In contrast, for the chief diffuse sources (urban road traffic, small industrial and heating systems), emissions were calculated with the availability of both opportune activity indicators for all the considered emission activities and pertinent emission factors, using literature data, i.e., AP-42 [15], an IPCC Guideline [16], and the EMEP/CORINAIR Emission Inventory Guidebook [17].

Because of the considerable impact of the road transport sector on total emissions in European nations, we conducted specific analyses for this sector, which depend on numerous factors: annual mileage, fuel consumption, driving patterns and climatic factors. Given these characteristics, emissions from the road transport sector were evaluated accurately using the COPERT method, which is a collection of algorithms and emission factors proposed at European level for the estimation of road traffic emissions [18,19]. The COPERT method permits the estimation of emissions for 230 vehicle categories grouped in five key groups [20]: cars, light and heavy duty vehicles, urban buses and coaches, and two-wheel vehicles. These are further subdivided based on fuel type, EU directives for emission limits, and engine displacement. Emissions can be calculated for 36 pollutants, including PAHs, dioxins, and potentially toxic metals contained in the fuel [21]. In the present paper, the COPERT procedure was used with a bottom-up approach, focusing on the municipal rather than national level; thus, we examined local variables regarding driving patterns, vehicle fleets, average trips, and fuel consumption. To evaluate in detail the environmental impact of road transport in the study area, the selection and gathering of these basic input data involved the following organizations and societies: the Italian Institute of Statistics; the Italian Automotive Association, which provided the number of registered vehicles and their composition at municipality level [22]; the Italian Institute for Environmental Protection and Research [23]; the Italian Association of Oil Companies, which provided the fuel sold for each Italian province in its annual oil market bulletins. The base year for the evaluation in the emission inventory was 2012.

### 2.2. Geochemical Characterization of Agricultural Soils

A large amount of data on soil, stream sediment and groundwater chemistry for the study area was produced in the last decade in the framework of various research projects. Activities related to the geochemical characterization of agricultural soils have been carried out aiming at the collection and elaboration of all geochemical data available from exploration activities already completed by the research group in charge of the associated sub-action [11,12,24,25,26,27,28].

Lima et al. [25] and Albanese et al. [24] determined for a large number (53) of inorganic elements a threshold value in soils to separate background concentrations from anomalies. The Multifractal Inverse Distance Weighted (MIDW) (available in the software GeoDAS [29]) was used for draw up the geochemical interpolated maps and to separate the background values from the anomalies using the separation-anomaly (S-A) method [30,31,32,33,34]. MIDW takes into account both spatial association and local singularity, preserving high frequency information, which is lost in any conventional moving average methods, such as kriging and ordinary IDW. The S-A method results in the production of two raster grid from the original MIDW maps; the two raster maps represent the spatial distribution of both potential anomalous values and baseline/background concentrations across a covered area. Basically, 2-D Fourier transformation can convert geochemical values into a frequency domain in which different patterns of frequencies can be identified. The signals with certain ranges of frequencies can be converted back to the spatial domain by inverse Fourier transformation and can be used to separate anomalies from background.

Further, to trace the origin and the extent of contamination in the environment, particularly useful has been the isotopic signature of geogenic and anthropogenic materials, in combination with concentration data for pollutants. Pb isotopic ratios (Pb^208^/Pb^206^, Pb^207^/Pb^206^) are used for discriminate the geogenic or anthropogenic origin of some Potentially Toxic Metals (PTMs) across the anomaly areas [11,12]. Lead isotopic ratios have been measured in selected soils on both leaches [using 1 M HNO_3_–1.75 M HCl (50:50)] and residues thereof.

For the lead isotopic analysis, samples were processed at USGS Radiogenic Isotope Laboratory in Reston (Washington, DC, USA); in a first step samples were sieved retaining 200 mg of the 60 mesh (250 μm) fraction and then dissolved by means of 3 mL of a solution containing 1.5 N HCL and 3 N HNO_3_. Samples were centrifuged for five minutes to separate the leachate from the solid residue. The residues were processed using a multistep treatment with the solution of HNO_3_, 4 N HCL and 0.5 N HBr. The leachates were digested in a microwave (Ethos Plus Microwave Lab station, USGS Radiogenic Isotope Laboratory in Reston, Washington, DC, USA) for 15 min and treated using the same above multistep process. All the samples (leachates and residues) were run through columns filled with anion exchange resin using 0.5 M HNO_3_ and 0.5 HBr as eluents [35].

Soil leachate reflects possible anthropogenic contamination, whereas soil residues indicate geogenic contributions. Results of the aforementioned studies indicated a significant impact of human activities on the environment. The distribution of PAHs in soils of the study area showed that PAHs are emitted from two main sources [24]: the burning of agricultural biomass, especially in the eastern part of the study area, and emissions from industries and heavy traffic near major urban settlements.

Data retrieved from earlier prospecting activities on soils [11,12,25], reporting concentrations for 53 elements (Ag, Al, As, Au, B, Ba, Be, Bi, Ca, Cd, Ce, Co, Cr, Cs, Cu, Fe, Ga, Ge, Hf, Hg, In, K, La, Li, Mg, Mn, Mo, Na, Nb, Ni, P, Pb, Pd, Pt, Rb, Re, S, Sb, Sc, Se, Sn, Sr, Ta, Te, Th, Ti, Tl, U, V, W, Y, Zn, Zr), were aggregated into a single database. The aggregation was possible since all the samples were analyzed by means of the same analytical method (ICP-MS following an *aqua regia* digestion) and they were processed at same analytical laboratory (ACME Analytical Laboratories Ltd., Vancouver, BC, Canada). For the sampling procedures and the quality of the data (accuracy and precision), see publications [11,12,25].

The database was used to generate a thematic cartographic set, including dot and multifractal IDW, interpolated maps for each of the analyzed elements, and a map of the contamination degree (CD) based on the 15 PTM’s considered by Italian Environmental Law (D. Lgs. 152/06). The CD map was produced through spatial analysis in a GIS environment, by dividing the multifractal IDW map of each element (predicted environmental concentration or PEC) by a reference concentration value (predicted no-effect concentration or PNEC) representing an equilibrium condition for the local environment. In our case, the PNEC was set in accordance with the concentration values assumed to be of geologic origin (background) for the area [25].

### 2.3. Aquifer Vulnerability to Contamination

The hydrogeochemistry of the study area is influenced by the inflows from adjacent carbonate aquifers, by the chemistry of the pyroclastic deposits and by saltwater intrusion near the Volturno River mouth (Figure 1). There are also mineralized areas along the borders of the Garigliano plain and the plain of the Volturno river—Regi Lagni (with CO_2_) and in the Phleagrean Fields GWB, due to the complex interaction between deep volcanic fluids, fresh groundwater and seawater [36]. In previous studies [37,38], for distinguishing anthropogenic and geogenic contamination in groundwater, natural background levels (NBLs) were assessed in part of the area. NBLs indicated that the contamination for As, F, Fe and Mn is often of geogenic origin.

Hydrogeological data (hydrochemical analysis, piezometric measures and stratigraphic data) were gathered from authorities, surveys and private companies. The stratigraphic succession was defined using data from boreholes (about 800). The piezometric pattern was obtained by interpolation of almost 1000 piezometric data gathered from 2001 to 2009 and updated in 2014 by targeted measurements in about 20 wells.

Hydrochemical data used for the present study (almost 350 sampling points) mainly refer to the main aquifer. In the southern part of the plain of the Volturno river—Regi Lagni GWB there is a larger dataset, related to arsenic and other organic and inorganic contaminants. In 2014, the dataset was integrated with about 50 new chemical analyses of groundwater sampled exclusively in the sectors more affected by contamination problems.

The map of susceptibility of the aquifers to contamination was derived from previously constructed maps, first standardized and then merged. All the previous maps were assessed using the SINTACS method [39], because it gives good results in alluvial aquifers and flat areas, counting on hundreds of applications.

This method evaluates vertical vulnerability using the seven parameters: depth to groundwater (S), recharge action (I), attenuation aptitude of the vadose zone (N), attenuation aptitude of the soil (T), hydrogeological characteristics of the aquifer media (A), hydraulic conductivity (C), and topographic slope (tS). Each mapped factor was classified into scores (1 to 10) that have an impact on pollution potential. Weight multipliers were then used for each factor to balance and enhance their importance. The final vulnerability index is a weighted sum of the score of the seven factors for the weights.

In the study area, the depth to the water table, is one of major factors affecting groundwater vulnerability, being the parameter unsaturated zone few vulnerable, apart a small belt along the coast, and the aquifer permeability homogeneous. Moreover, parameters referring to the territory as slope, recharge, etc. were already considered in the multi-criteria analysis. For these reasons, for the multi-criteria analysis, we preferred to use only the depth to water to represent the vulnerability, in order to avoid redundancy using a layer derived from numerous layers, even because attempts made using the whole vulnerability map gave very similar results. The new map called “minimum depth to water” (S_min_) has been drawn up in the shallow unconfined aquifers as the depth of the water table (evaluated from the DEM and that surface) and in the confined (or semi-confined) aquifer as the depth of the bottom of the Campanian Ignimbrite confining layer (evaluated from stratigraphic data).

### 2.4. Soil Hydraulic Characterization of the Study Area

Especially for occasionally irrigated but mostly rainfed plants, such as those commonly used into phytoremediation protocols, the knowledge of the maximum amount of water that a soil profile is able to store is a decisive factor for designing successful bioremediation actions in a contaminated area. This information is suitably embedded in the well-known “available water” (AW) parameter, which is the difference in the volumetric soil-water contents between the field capacity (FC) and permanent wilting point (PWP) conditions in soil (i.e., AW = FC − PWP) [40,41]. In the cases of relatively large land areas, such as the study area considered here, mapping the spatial variations of AW is an effective dynamic indicator to evaluate the efficiency of the selected phytoremediation protocol in the different parts of the territory.

We performed the calculation of AW through two different approaches. The FC value is determined dynamically using a Richards-based numerical model as the average volumetric water content remaining in a soil profile, after having been completely wetted with water and free drainage beyond the root zone has become negligible [40,41]. Instead, the PWP value is estimated using the pedo-transfer function (PTF) of Vereckeen [42]. A PTF is a regression equation that uses soil texture, soil organic carbon content and oven-dry bulk density as input information and provides as output the parameters that describe the soil hydraulic properties (i.e., the water retention and the hydraulic conductivity functions). The PWP value is determined from the soil-water retention function as the volumetric soil-water content at the matric suction of 1500 kPa.

For this purpose, soil characteristics were determined in numerous locations of the study area and the experiments were conducted in the laboratory on undisturbed soil cores. To plan a cost-effective and efficient sampling strategy and select representative sampling sites, we used partly our already available soil database and partly the pedological studies “Land Systems Map of Campania Region” [43] and “Soil Map of the Province of Napoli” [44].

The standardized map, produced for the Spatial MultiCriteria Decision Analysis, was based on the dimensionless index AW_index_, defined in each location as follows:

AW_index_ = (AW − AW_min_)/(AW_max_ − AW_min_) (1)

where AW_min_ and AW_max_ are the most common minimum and maximum values for AW found in the literature [45].

### 2.5. Spatial MultiCriteria Decision Analysis

For the multi-criteria analysis, data from the other groups have been mainly used. The dataset was completed with additional data sources [46].

All layers were organized according to a criteria tree, divided in “constraints” and “general factor”. Constraints (Protected areas, artificial surfaces, etc.) are binary raster, with positive-negative polarity. Factors were divided in three sub-categories of raster dataset: Water (depth to groundwater and availability of water Index), Soil (baseline values for four geogenic chemical elements) and Territory (RUSLE erosion map, land cover map reclassified, radon-prone areas, distance from natural areas, NIPS areas, digital elevation model height and slope, distance from roads and distance from artificial surfaces). The factors are non-binary layers [47]. All raster maps were standardized [48] and weighted through the ANP technique [49] and pairwise comparison thanks to the spatial multicriteria evaluation module of ILWIS 3.8. Air component and human health data were not considered.

From the S-MCDA is sorted out the suitability map along with the identifications of areas more susceptible to land use change. The suitability map was classified in 6 classes with Natural Jenks classification [50]. From the S-MCDA is sorted out the suitability map consistent with the bio-remediation techniques. The identifications of the areas more susceptible to land use change is a consequence of suitability map, with a raster ranking. The suitability map was classified in 6 classes. Firstly, areas with natural or anthropic constraints were highlighted. The other classes were sorted out from a Natural Jenks classification [50], which group data into like classes, minimizing differences within each data class and maximizing differences between classes. The classification has to be considered as a baseline from which to build scenario analysis [48].

### 2.6. Human Exposure and Health Assessment

In developed countries, a significant decrease in Standardized Mortality Rates (SMR), particularly from neoplastic diseases, has been observed in recent years. In Italy, for example, a 22.8% decrease of SMR for all tumours has been recently reported. However, in the Campania Region, this decrease has been just 14% [51]. Further, in the provinces of Napoli and Caserta (Figure 1), SMR have shown an increase by as much as 20% for some tumour types [52]. Similar results emerged from the “SENTIERI” study, analyzing SMR for the main causes of death (mainly cancers and cardiovascular diseases), published by the national Higher Institute of Health [51].

The increase in SMR for tumours of the study-area population has been associated, directly or indirectly, with exposure to chemical, physical or biological pollutants from repeated illegal dumping of waste in superficial or underground landfills. Although a causal relationship between environmental exposure and health effects has not yet been convincingly demonstrated, a possible association has been discussed or suggested by a number of publications [51,52,53,54,55,56,57,58,59,60]; see details in the supplementary material.

In this study, the exposure of the population was determined using databases resulting from some environmental matrices, namely those on soil and on groundwater [24]. For human exposure risk analysis, we used the GIUDITTA Programme. This was developed by the Province of Milan, based on the Risk Based Corrective Action (RBCA) procedure reported by the American Society for Testing and Material (ASTM) and adopted by the Italian National and Regional Agencies for Environmental Protection (ISPRA-ARPA). This procedure is based on a graded approach including three levels of analysis, each progressively more detailed. The use of the second and third levels of analysis requires more information and increasingly more accurate characterization of the area. The first and second levels of analysis were executed for human exposure to both toxic and/or carcinogenic chemicals: for carcinogens in the absence of a threshold dose, risk is represented by the probability of additional tumour cases in a lifetime, while for toxic non carcinogenic chemicals, risk is calculated by considering a threshold dose.

Level 1 risk analysis (1-RA) was performed for all contaminants, whereas level 2 risk analysis (2-RA) was only done for chemicals exceeding the legal limit indicated by Italian law (D. Lgs. 152/06). Since soil and groundwater samples were collected separately at different locations and times, the risk analyses had to be executed separately for the two materials. The level 2 analysis could be performed differently, depending on the number of data/samples available for each chemical: using the 95th percentile for chemicals for which there were sufficient data and using the average concentration for chemicals with limited data.

The health assessment of the population living in the study area was performed using the ISTAT mortality database (mortality study) and data from hospital discharge sheets (SDO) (morbidity study) collected by the regional health authority of the Campania region (ARSAN) for the periods 2006–2011 and 2003–2011, respectively. The mortality data provided by ISTAT (http://www.istat.it/) included the following information: residence in the Campania region (the data distribution by individual municipalities, although important, was not provided because of privacy protection rules), age in 5-year classes (0–4, 5–9, …, 80–84, 85+), and cause of death.

For the morbidity study, we assessed the distribution of malignant tumours for Campania residents discharged from any hospital in Italy during 2003–2011 with a diagnosis codified according to ICD-9 codes (from 140 to 208). For each patient, the following information was recorded: patient code, date of hospital admission, major diagnosis (site and type of tumour), code of residence, gender and age. To identify incident cases and date of diagnosis, the first admission to hospital in chronological order was considered for each patient. SMR for all causes and individual types of tumour were calculated for the 77 municipalities together and compared with those of the entire region for each year. The direct standardization method was used, based on the Campania Region population registered in 2011 (http://demo.istat.it/), to correct for differences in the age distribution of the population between the study area and the Campania region.

## 3. Results and Discussion

### 3.1. Air Pollutants and Emissions

Global air emissions evaluated for all the other SNAP Sectors in the Litorale Domizio-Agro Aversano area for 2012 are given in Table 1 and Table 2. These tables provide emission levels of the main air pollutants (CO, VOC, NO_X_ and PM_10_) and TPMs emitted from diffuse and point sources, expressed in tons and kg per year, unbundled for CORINAIR sectors of activity.

Examination of these results clearly shows that for the study area, a significant share of total emission levels is from the road transport sector. As in other provinces of southern Italy, renewal of the vehicle fleet is much slower than in other parts of the country, with clear consequences for emission levels of the pollutants.

Reviewing the contribution of other sectors to total emissions, CO is mostly emitted by the road traffic sector. For NO_X_ emissions, non-road traffic (other mobile sources and machinery) was the major emission sector. The tendency of total PM_10_ emissions in the area is controlled by an equilibrium between declining emissions from the combustion of oil and coal in the residential sector and a rising share of emissions from diesel cars, owing to an increase in sales and use of such vehicles [61].

**Figure 4:**
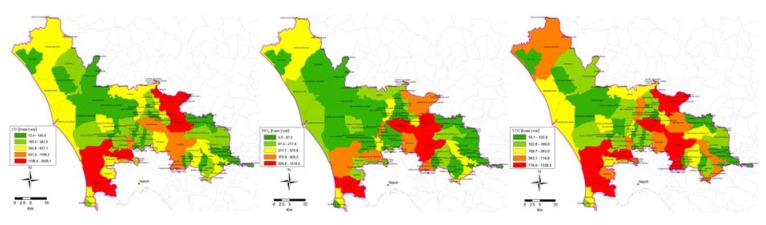
Distribution of CO, NO*_x_*, VOC total emission, with spatial disaggregation at municipal level.

With regard to TPMs, Table 2 clearly shows that the pollutants copper, nickel and lead are emitted in greater quantities than other TPMs in the area. For these TPMs, a considerable share is from road traffic. To summarize the chief results of the other sectors, it is evident that Ni and Pb are mainly emitted by industrial sources burning oil, natural gas or LPG. The major results of the present study related to point and non-point sources were collected in the GIS environment to construct maps of emission levels, with geographic disaggregation by municipality. These results were obtained by summing emission levels by pollutant for each municipality in the area. Total levels of the major air pollutants were disaggregated by municipality in relation to the emission inventory for the entire area (Figure 4). Regarding spatial shares of total emissions, maximum levels have been localized around Caserta, where the principal sources of air pollution are as follows:

- The road traffic sector, given the dense networks of roads and motorways in the area.
- Industrial facilities burning oil, coal and natural gas. These plants are largely in the industrial area of Caserta and mostly emit CO, NO*_x_* and PM_10_.

Validation of the air pollution results was done by reference to real measurements, which were mainly conducted near main urban intersections. Air quality data were obtained from the regional information system, which contains data on air quality monitoring networks and stations.

### 3.2. Agricultural Soils and Groundwater Characterization

The soil analyses results support the following statements:

- Al, As, Ba, Be, Fe, La, Th, Ti, Tl, and U, generally highly concentrated in the volcanic soils of the Phlegrean Fields, Mt. Roccamonfina volcano area (northern part of the study area) and Nola-Pomigliano area (north of Mt. Somma-Vesuvius volcano), are mostly of geogenic origin.
- Co, Cd, Cr, Fe, Mg, Mn, Ni, Sc and V, concentrated in the interior of the study area, across the Volturno River plain (in certain strongly anomalous areas), and along the coastline south and north of the river mouth, are likely related to the co-precipitation effect induced by Fe and Mn hydroxides in soil of alluvial origin.
- Sb, Pb, Sn, Zn, Cd, Hg and Cu, generally located at the Volturno River mouth and certain urban centres across the study area, may be historically linked to both heavy motor vehicle traffic and industrial settlements.
- Sr, K, Na, Ba and P enriched in volcanic soils north of Mt. Somma-Vesuvius are probably dependent on the natural composition of volcanic sediments and their extensive agricultural use.
- Se, B and S found in considerable concentrations in the Nola area, across the Phleagrean Fields, and in a large area south of the Volturno River mouth, may be related to hydrothermal phenomena.

At many sites, some of the PTMs reported above (As, Be, Cd, Cu, Pb, Zn, Tl and Zn) had concentrations in soils exceeding the corresponding thresholds established by Italian environmental law (D. Lgs. 152/06), suggesting the need for detailed scientific investigation to assess the risk from this medium to the exposed human population, for example:

- Around 10% of analysed samples of soils exceeded the thresholds for As in the case of residential/recreational land use (20 mg/kg), including two sites where As even exceeded thresholds for commercial/industrial land use (50 mg/kg).
- Around 20% of analysed soils had Cu concentrations greater than thresholds (120 mg/kg for residential/recreational and 600 mg/kg for industrial/commercial land uses), especially in the Nola-Pomigliano area where values reached 677 mg/kg and the use of Cu-rich compounds in agriculture cannot be excluded as a potential contamination source.

**Figure 5:**
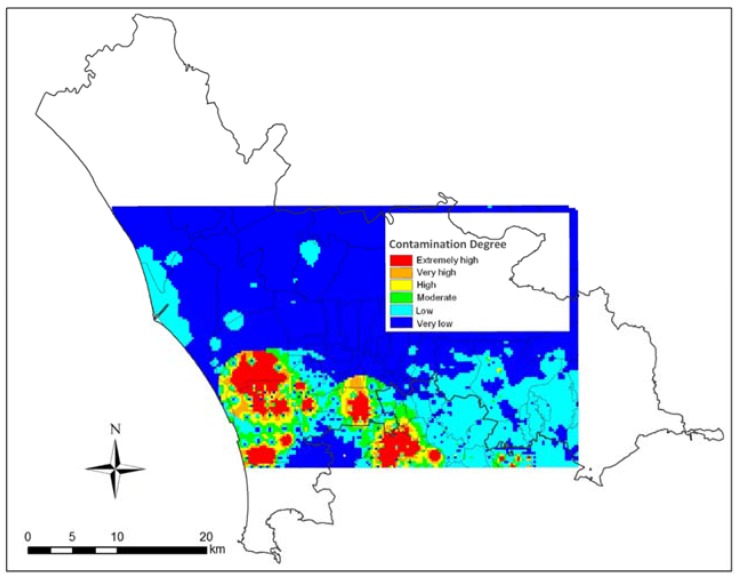
Map of inorganic contamination of soils in the central part of the study area, based on the distribution of 15 potentially toxic metals.

The CD map (Figure 5) is based on the distribution of As, Be, Cd, Cr, Co, Cu, Hg, Ni, Pb, Sb, Se, Sn, Tl, V, Zn. Figure 5 represents a part of the study area and itshows that the areas in red colours are the most polluted of the entire study area.

**Figure 6:**
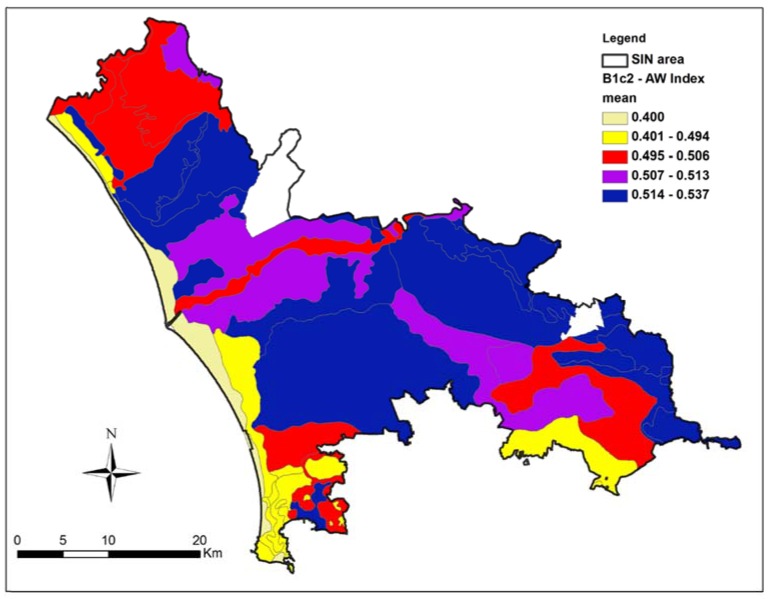
Map of Available Water index (AW_index_) in soils.

It has to be stressed that site specific studies are needed case by case, to establish clearly the natural or anthropogenic sources of the above reported metals/metaloids. Regarding soil hydraulic parameters, a map of AW_index_ is reported in Figure 6 and it shows greater or lesser allocation of AW in dimensionless terms.

The hydrogeochemical groundwater characterization (cartography available at the website http://www.ecoremed.it) shows very clearly the variable contexts in the area. Nitrate is the most present contaminant, often exceeding the 50 mg/L threshold (D. Lgs. 152/06). High values of Fe and Mn near the Volturno River and Acerra are caused by reducing conditions and mineral waters, which also produces high concentrations of SO_4_. Elsewhere, isolated maxima of Fe and Mn, often combined with organic and inorganic contaminants, are probably of anthropogenic origin and must be carefully considered. Contamination by fluoride and As has a geogenic origin, closely related to volcanic and pyroclastic rocks (Roccamonfina volcano, Phlegrean Fields and Vesuvius).

**Figure 7:**
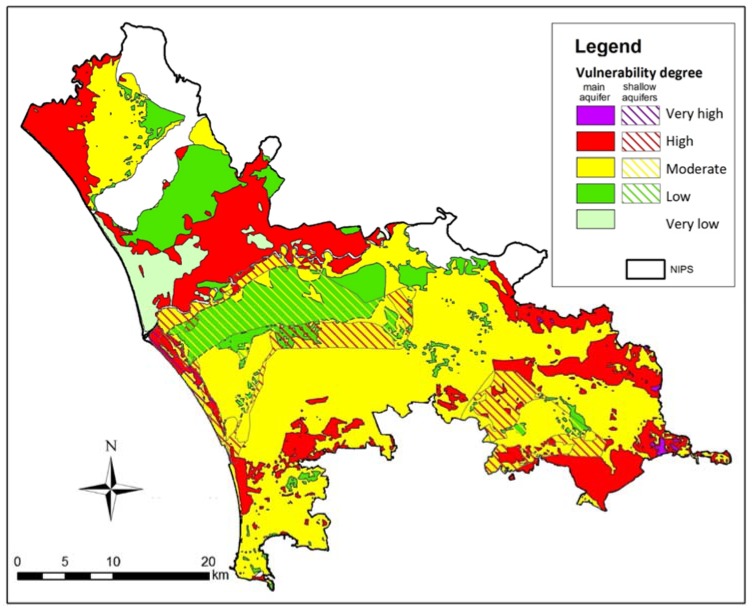
Map of susceptibility of aquifers to contamination.

The map of susceptibility of the main aquifers to contamination (Figure 7) shows the prevalence of moderate vulnerability, with areas of low permeability near the Volturno River, where clay and peat are widespread. In the coastal areas, vulnerability is substantial for small depths to water and for the grain size of the vadose zone. Strong vulnerability is also evident at the foot of carbonate mountains and where the aquifer is semi-confined. The map of susceptibility of the shallow aquifers to contamination shows greater vulnerability because of the small depth to water.

The map of minimum depth to water (S_min_) considered in the S-MCDA (see Section 2.3) merges the above aspects and indicates the area close to the Volturno River as very protected from contamination, confirming a natural source of high Fe and Mn there.

### 3.3. Suitability Map of the Bio-Remediation Reclamation Techniques

The criteria map layers used in this study were divided in four constraints map and 12 factor maps. The constraint raster dataset delimitated portion of study area excluded from the application of new crops for phytoremediation purpose given a value of 0. The remaining areas are made available for consideration with a value of 1. In the factor raster data set, which displays a variable degree of suitability consistent with dataset analyzed, factors weights were assigned in two phases. The computed Factor Weights (FW) are reported in the Table 3, Table 4 and Table 5. The multiplications of the factors weights each other outputted three intermediate results (Table 6) [62].

The three intermediate maps were aggregated to give a single composite index map, showing varying degree of suitability [62]. According to the quality level assessed for each environmental matrices, the study area could be described by a set of homogeneous zones, basing on its Environmental Quality Standards (EQS) reported in Table 7.

**Figure 8:**
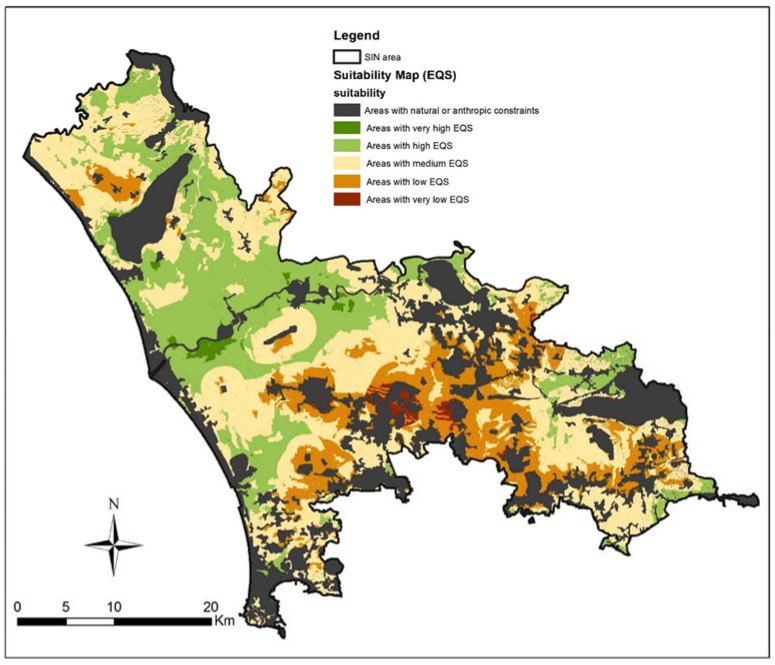
Suitability map referred to areas suitable for bioremediation reclamation techniques (from medium to very low Environmental Quality Standards).

The final map was grouped into six categories ranging from “constraints” to “very high EQS” (Figure 8). Natural or anthropic constrains represents the 29% of the total area. The map shows that the 22% of the study area has high EQS and less than 1% of the study area has a very high EQS. Nevertheless the half part of the study area is susceptible of a land use change and so it is suitable for the application of bio-remediation reclamation techniques. As a matter of fact the 34% of the total area has a medium EQS, the 13% has a low EQS and less than 1% has very lowEQS (Figure 8).

These classes have established a negative match in terms of land susceptibility to land use change (LUC), so were recognized as “very high LUC suitability” areas with “very low EQS”; were classified as “high LUC suitability” the areas with “low EQS”; consistently areas with “medium LUC suitability” were sorted out from “medium EQS”. Because land-use change in the area could be impelled by different driving forces, a different range of applicability for the reclamation activities was developed from generated suitability maps.

Land use and related employment opportunities are key to the future development of the study area. Socio-economic deprivation indices there are the worst in Italy (39% of families are in a condition of deprivation) and it is well known that they represent another risk factor, for both human health and for antisocial behaviour that can cause pollution such as illegal waste disposal and burning [63,64,65,66].

One of the main limitation in the method is the difficulty of transferring in graphical form, data protected by privacy laws. In the case of health data, the frequency/area ratio resulted not statistically significant, and so it was not possible to analyze within the framework of S-MCDA. Another criticism is the difficulty of collecting data distributed uniformly for the whole study area. These constraints have limited the potential of the tool but not prevent the implementation of new advancement in the research. The framework can be implemented as it is dynamic, flexible, transparent and allows the calibration of scenarios sized on specific needs of the area examined.

### 3.4. Human Exposure and Health Assessment

The results of this part of the study are reported here in two sections: (a) assessment of risk to human health from exposure to contaminants in soil and water, based on data indicated at (Section 2.6); and (b) an epidemiological study, based on comparison of mortality and morbidity data of the study area population with those of the entire Campania region.

#### 3.4.1. Human Exposure

For inorganic chemicals in soil, 1-RA (Section 2.6) showed high levels of some contaminants, exceeding the limit for Pb, Cu, Zn, As, V, Tl, Se, Sn and Be. Cadmium, Sb and Cr (CrVI) concentrations were all below limits and, therefore, for these chemicals and Se (for which the 95th percentile value was below the limit), a 2-RA (Section 2.6) was not done. 2-RA gave unacceptable RF (risk factor) levels for As and Be. Both these chemicals, however, are likely to be geogenic. For organics, 1-RA showed that nearly all samples exceeded the limits for residential areas (C < 12, C > 12, dichloromethane, indenopyrene, and DDD/DDT/DDE) and, in some cases, for commercial areas (3% of samples for C > 12, 33% of samples for DDD/DDT/DDE). This suggests the presence of a widespread anthropogenic activity. 2-RA also gave results exceeding acceptable levels for certain chemicals. For dichloromethane and indenopyrene, 2-RA for carcinogenic effects showed unacceptable RF values for residential areas. Nearly all samples exceeded the limits for C < 12 and C > 12 hydrocarbons. Nevertheless, 2-RA for non-carcinogenic effects at the 95th percentile value showed for both these chemical groups levels of risk index (RI) for hydrocarbons within acceptable levels, for both adults and children. In some sectors, contaminants in groundwater (As, Se, Pb, and ammonium ion) were above the limits according to 1-RA.

In summary, 1-RA performed for organic and inorganic chemicals measured in the two matrices (soil and groundwater) revealed concentrations above the current limits stated in Italian law, for several toxic and carcinogenic chemicals in both matrices, although these limits should not be applied to geogenic chemicals. 2-RA performed on chemicals that exceeded the 1-RA limit showed mainly acceptable values, but also some unacceptable ones (As, Be, dichloromethane and indopyrene in soil).

#### 3.4.2. Health Assessment

Results of the epidemiological study are reported below for the mortality and morbidity studies separately. Tumour Registry data are available for most but not all the municipalities of the study area and, therefore, we used, as surrogates for the mortality and the morbidity studies, the data available from the ISTAT mortality database and from hospital discharge sheets (SDO), respectively.

*Mortality study.* Mortality for all causes over 2006–2011 in the study area was generally higher than that of the entire Campania region (SMR = 106 and 108 in males and females, respectively). In particular, in males, an excess was observed for all tumours, cardiovascular, respiratory, digestive and endocrine-metabolic-immune diseases, and in females for all tumours, cardiovascular, endocrine, digestive and endocrine-metabolic-immune diseases. Regarding neoplastic diseases, an excess for stomach, colon-rectum and liver was observed in both males and females, and for lung and bladder in males. For details on mortality data see the supplementary material.

*Morbidity study*. Analysis of the hospital discharge sheets (SDO) for neoplastic diseases in the same period (2006–2011) showed that the percentage of hospital admissions for tumours against all admissions was similar in the study area and the entire region for both males and females. This percentage was greater for males than females in both areas, as expected. For all tumours together, a statistically significant excess of hospital admissions was observed in the study area for males but not for females (SMR = 103 and 100, respectively), with rates decreasing with time for both males and females. When single organ sites are considered, in residents of the study area relative to the entire region a statistically significant excess was observed for lung (in males), colon-rectum (in females), liver, stomach (decreasing with time in males) and breast, while prostate registered fewer admissions.

No statistically significant excess or trend with time was observed for bladder in males, nor for non-Hodgkin lymphoma or leukemia in males or females (except for a decrease in leukemia in females). Finally, fewer admissions were observed for the uterus (corpus and cervix), with significantly smaller values for the study area as compared with the entire region (see the Supplementary Materials).

It is difficult to determine whether the two observations are linked, i.e., the presence of contaminants at concentrations in soil and groundwater above current health-based limits on the one hand and the higher mortality and morbidity rates for various neoplastic diseases in the population on the other,. A number of limitations hampered the interpretation and especially the assessment of a potential association between the two findings. These limitations were the absence of actual exposure data in the population (biomarkers), a lack of information on the main confounding factors present in the population (smoking habits, diet, socio-economic status and deprivation), (long) lag times between any relevant exposure and onset of disease, difficulties in assessing any cumulative, potentiating or synergistic effects from multiple chemicals, and others. Future research work investigating the potential association between exposure and health effects should attempt to overcome these limitations, in particular by targeted, chemical-specific biological monitoring.

## 4. Conclusions

The environmental characterization of the area presented in this paper, based on a synergistic approach among different scientists, allowed the evaluation of cumulative effects of the environmental features (air, soil, soil hydraulic and groundwater), considering combination and super position effects.

Beginning from the collection of existing data on all environmental factors, systematized in a GIS inventory, the study evaluated the contribution of each to overall chemical contamination, bearing in mind the potential effects on human health. Afterwards, we integrated the GIS with the MultiCriteria Decision Analysis (MCDA) to perform the Spatial MultiCriteria Decision Analysis (S-MCDA), that allowed the evaluation of the cumulative effects of all the environmental features in the study area. Finally, the S-MCDA was used to draw up the “suitability map”, indicating the sectors consistent with bio-remediation reclamation techniques and the consequent identification of the areas more susceptible to land use change. The individual examination of the environmental features make it possible some more conclusions:

- The atmospheric data indicated that a significant part of total emissions was from road traffic. The spatial distribution of emission levels depicts maximum emission densities around the city of Caserta.
- In many agricultural soils, Potentially Toxic Metals (PTMs) were found in concentrations exceeding the legal limits for soil established by Italian and European environmental laws. Some elements (As, Be and Tl, and possibly Sn) are of geogenic origin, Cd is related to the effect of co-precipitation induced by the presence of Fe and Mn in soils of alluvial origin, and others (Cu, Pb and Zn) are related to both heavy motor vehicle traffic, industrial settlements (Pb, Zn) and agricultural practises (Cu to vineyards).
- The hydrogeological study, concerned an assessment of groundwater quality and the susceptibility of aquifers to contamination, shows that in many cases the presence of high values of F, As, Fe and Mn is of geogenic origin and the depth to water is the most significant parameter influencing the groundwater contamination (mainly by nitrates of agricultural origin).
- The soil hydraulic characterization allowed the drawn up of the Available Water (AW) index map, which represents water stored in the soil profile and available for plant growth, which was an useful layer for the “suitability map”.
- The human exposure and health assessment parts of the study gave two sets of results, both of which may be of potential relevance to public health. The epidemiological study provided new evidence confirming higher rates of certain diseases in the population of the study area relative to those of the entire Campania region. In an epidemiological observational study such as this, any exclusion of specific chronic degenerative diseases, including cancer, may lead to selection bias, resulting in a likely over- or underestimation of risk, and this is why all types of disease or cancer should always be assessed. As to the potential confounding factors may indeed play a significant role, nevertheless this information is simply not available in the existing databases. Despite this limitation the data reported in the study are original and important to focus future research.

Finally, no definite conclusions can be drawn as regards the possible relationship between presence of contaminants in the environment and health conditions of the population, also influenced by the precarious socio-economic conditions of the population in the study area. Indeed, deprivation indices are known to be strongly correlated with various risks to human health, including mortality and morbidity.

In the study area, the role of the land management and the landscape planning policies assumes great importance, as does the reduction of poverty and deprivation of the population. Moreover, an in-depth knowledge of the physicochemical conditions of the area can support the decision-makers to work toward more sustainable and effective planning of policies, measures and operational works.

From a methodological point of view, the present study encourages the application of the used approach, that increases the possibilities for studying the combined effect of more environmental factors. The novelty of the approach is to combine the environmental features by means the S-MCDA method and to assess the health risk, that lead to an integrate approach for the analysis of the problem and for the application of new management policy.

## Figures and Tables

**Table 1:** Total annual emissions of major air pollutants in the Litorale Domitio-Agro Aversano area, disaggregated by CORINAIR sectors.

SNAP Sector	CO		COV		NO*_x_*		PM_10_	
	(t)	%	(t)	%	(t)	%	(t)	%
Combustion in energy and transformation industries	2.3	0.01%	3	0.02%	250.9	1.72%	4.1	0.16%
Nonindustrial combustion plants	1092.5	3.54%	104.3	0.54%	482.8	3.31%	280.3	10.89%
Combustion in manufacturing industries	1260.4	4.09%	164.2	0.86%	2984.2	20.47%	202.7	7.88%
Roduction processes	478.5	1.55%	818.5	4.26%	508.2	3.49%	383	14.88%
Extraction and distribution of fossil fuels/geothermal energy	0	0.00%	275.1	1.43%	0	0.00%	0	0.00%
Solvent and other product use	0	0.00%	7780	40.53%	0.6	0.00%	0.7	0.03%
Road transport	25,714.1	83.41%	7846.5	40.87%	4054.5	27.81%	1006.3	39.10%
Other mobile sources and machinery	2109	6.84%	975.9	5.08%	6292.6	43.16%	677.2	26.31%
Waste treatment and disposal	47.9	0.16%	432.2	2.25%	5.7	0.04%	9.9	0.38%
Agriculture	45.1	0.15%	698.7	3.64%	0.8	0.01%	4.7	0.18%
Other sources	78	0.25%	98.5	0.51%	0	0.00%	4.6	0.18%
Total	30,827.8	100%	19,196.8	100%	14,580.4	100%	2573.5	100%

**Table 2:** Total annual emissions of PTMs in the Litorale Domitio-Agro Aversano area, disaggregated by CORINAIR sectors.

SNAP Sector	As		Cd		Cr		Cu		Hg		Ni		Pb	
	(kg)	%	(kg)	%	(kg)	%	(kg)	%	(kg)	%	(kg)	%	(kg)	%
Combustion in energy and transformation industries	0.1	0.05%	0.1	0.09%	0	0.00%	0.1	0.01%	0	0.00%	0.1	0.01%	0.5	0.02%
Nonindustrial combustion plants	5.6	2.52%	7.2	6.28%	14.1	4.01%	7.2	0.68%	2.3	2.06%	197	20.30%	8.2	0.36%
Combustion in manufacturing industries	215	97.07%	101	87.78%	201	57.15%	45.9	4.34%	109	97.40%	572	58.99%	1882	81.71%
Production processes	0	0.00%	0	0.00%	107	30.32%	2	0.19%	0	0.00%	158	16.31%	7	0.30%
Extract. and distrib. of fossil fuels/geothermal energy	0	0.00%	0	0.00%	0	0.00%	0	0.00%	0	0.00%	0	0.00%	0	0.00%
Solvent and other product use	0	0.00%	0	0.00%	0	0.00%	0	0.00%	0	0.00%	0	0.00%	0	0.00%
Road transport	0	0.00%	4.7	4.10%	23.7	6.73%	806	76.24%	0	0.00%	33.1	3.41%	380.2	16.51%
Other mobile sources and machinery	0.6	0.27%	1.8	1.57%	6	1.71%	195	18.47%	0	0.00%	8.6	0.89%	19.6	0.85%
Waste treatment and disposal	0.1	0.05%	0.2	0.17%	0.3	0.09%	0.8	0.08%	0.6	0.54%	0.8	0.08%	5.7	0.25%
Agriculture	0	0.00%	0	0.00%	0	0.00%	0	0.00%	0	0.00%	0	0.00%	0	0.00%
Other sources	0	0.00%	0	0.00%	0	0.00%	0	0.00%	0	0.00%	0	0.00%	0	0.00%
Total	222	100%	115	100%	352	100%	1057	100%	112	100%	969	100%	2303	100%

**Table 3:** Comparison matrix for Environmental Characterization—WATER (ECW).

	S_min_	AW_index_	NW
S_min_	1		0.5
AW_index_	1	1	0.5

S_min_ = Depth to groundwater; AW_index_ = Avaibility of Water Index; NW = Normalized Weights.

**Table 4:** Comparison matrix for Environmental Characterization—SOIL (ECS).

	SnB	BeB	TlB	VB	NW
SnB	1				0.10
BeB	3	1			030
TlB	3	1	1		0.30
VB	3	1	1	1	0.30

SnB = Baseline value Sn; BeB = Baseline value Be; TlB = Baseline value Tl; VB = Baseline value V; NW = Normalized Weights.

**Table 5:** Comparison matrix for Environmental Characterization—TERRITORY (ECT).

	Er	LC	Rad	PAd	Rd	Ad	NW
Er	1						0.062
LC	3	1					0.187
Rad	3	1	1				0.187
PAD	3	1	1	1			0.187
RD	3	1	1	1	1		0.187
AD	3	1	1	1	1	1	0.187

Er = Erosion; LC = Land Cover; Rad = Radon; PAD = Protected Areas Distance; RD = Road Distance; AD = Artificial surface Distance; NW = Normalized Weights.

**Table 6:** Comparison matrix for Environmental Characterization—Intermediate Factors.

	ECW	ECS	ECT	NW
ECW	1			0.33
ECS	1	1		0.33
ECT	1	1	1	0.33

ECW = Environmental Characterization—WATER factor; ECS = Environmental Characterization—SOIL factor; ECT = Environmental Characterization—TERRITORY factor; NW = Normalized Weights.

**Table 7:** Suitability value for the Environmental Quality Standards EQS classes.

Environmental Quality Standards (EQS)		Suitability Value (0–1)
1	Areas with natural or anthropic constraints	0.00–0.01
2	Areas with very low EQS	0.01–0.58
3	Areas with low EQS	0.58–0.64
4	Areas with medium EQS	0.64–0.69
5	Areas with high EQS	0.69–0.74
6	Areas with very high EQS	0.74–0.76

## Supplementary Materials

The following are available online at www.mdpi.com/1660-4601/14/7/693/s1, Table S1: Recent epidemiological studies addressing or suggesting the relationship between environmental risk factors and human health in the Campania Region.
