# Supplementary material for: An Integrated Approach for the Environmental Characterization of a Wide Potentially Contaminated Area in Southern Italy
Source: Int J Environ Res Public Health. 2017 Jun 27;14(7):693. doi: 10.3390/ijerph14070693 (PMC5551131; doi:10.3390/ijerph14070693)
Supplement: Supplementary file 1 [file ijerph-14-00693-s001.pdf]

Supplementary Material

Table S1. Recent epidemiological studies addressing or suggesting the relationship between environmental risk factors and human health in the Campania Region

First author & year of publ.	Study design	Data sources	Outcome	Summary of findings
Crispo A et al., 2013	Geographical study	ISTAT, Naples and Caserta provinces	Trend of mortality (APC) 1988-2009	The decline observed in SMR between 1998 and 2009 in men of Naples and Caserta provinces was less marked compared with males from Italy overall. Focusing on men aged 65 and older, the decline in cancer mortality found for Italy overall diverged from the slight increase emerged from the metropolitan area of interest.
Barba M et al., 2011	Review	Scientific evidence on waste exposure and health outcomes in Campania region	Cancer mortality (overall and site specific) and congenital malformations	Association between waste exposure and health outcomes in specific areas of Campania Region, where improper waste management and illegal waste trafficking have been documented.
SENTIERI Project ISS, 2014	Geographical study	ISTAT 77 Comuni Campania Region (SIN)	All-cause mortality, cancer mortality	Significant SMR and adjusted for deprivation (SMR ID) for the main causes of death (cancers, cardiovascular diseases, etc.)
Musmeci L et al., 2010	Geographical study	The Regional Agency for Environmental Protection (ARPAC) has identified and characterized the various authorized/ unauthorized dumping sites.	A synthetical-waste index (SWRI)	Municipalities along the border between the provinces of Naples and Caserta and located on the Tyrrhenian coast are exposed to higher waste-related health risk.
Martuzzi M et al., 2009	Geographical study	Mortality data from ISTAT (1994-2001) for 196 municipalities of the prov. of Naples and Caserta	All-cause mortality, cancer mortalities and congenital anomalies	Significant excess relative risks (ERR, %) for all-cause mortality in men and women; increased risks in both genders for all cancer mortality and liver cancer; significantly increased risks for stomach and lung cancer in men only. Significant ERRs also found for malformations of the urogenital system and of the central nervous system.
Fazzo L et al., 2008	Geographical study	ISTAT, Naples and Caserta provinces (1994-2001)	Cancer mortality, congenital anomalies	Significant clustering of mortality by specific cancers (lung, liver, gastric, kidney and bladder) and prevalence of total and specific malformations in the northern part of Naples and southern part of Caserta provinces.
Comba P et al., 2006	Geographical study	ISTAT, Naples and Caserta provinces (1994-2001)	Distribution of cancer mortality and birth defects in the Provinces of Naples and Caserta	Significantly increased rates of all cause-mortality, cancer mortality and congenital malformations in the southeastern part of the province of Caserta and in the northwestern part of the province of Naples.
Menegozzo M et al., 2008	Dossier	ARPAC and IZSM dat (Environment and food monitoring) Provinces of Naples and Caserta	Dioxin's risk in the Municipalities of Campania	Soils exceeding the regulatory limit for dioxins were found in 5 Municipalities (one sample). In 45 Municipalities livestock used for food production exceeds the limits (one or more samples for each Municipality).

WHO, CNR, ISS, Regione Campania 2008	Waste processing: impact on human health	Data organized in a GIS (dal 2004)	Development of synthetic indicators of waste danger (IRSC)	Agreement with the results of epidemiological studies (already carried out) and the geographical distribution of the IRSC at municipality level.
De Felipe, Di Domenico A (Ed.) 2010. SEBIOREC Final Report	Epidemiological study of biomonit. (2007-2010)	Individual blood sample (male+female), and 62 milk samples from primiparous.	Exposure to several organic and inorganic contaminants in biological samples from healthy donors	Different results: significant and /or not significant according to organic and inorganic contaminants (see pag. 47-63)
